# Canine Adenovirus 1 Isolation Bioinformatics Analysis of the Fiber

**DOI:** 10.3389/fcimb.2022.879360

**Published:** 2022-06-13

**Authors:** Ben Wang, Minchun Wang, Hongling Zhang, Jinfeng Xu, Jinyu Hou, Yanzhu Zhu

**Affiliations:** ^1^ Animal Science and Technology College, Jilin Agriculture Science and Technology University, Jilin, China; ^2^ Institute of Special Animal and Plant Sciences, Chinese Academy of Agricultural Sciences, Changchun, China; ^3^ College of Veterinary Medicine, Jilin Agricultural University, Changchun, China

**Keywords:** canine adenovirus 1, phylogenetic tree, bioinformatics analysis, B cell epitope, T cell epitope, *Fiber*

## Abstract

Canine adenovirus type 1 (CAdV-1) is a double-stranded DNA virus, which is the causative agent of fox encephalitis. The *Fiber* protein is one of the structural proteins in CAdV-1, which mediates virion binding to the coxsackievirus and adenovirus receptor on host cells. The suspected virus was cultured in the MDCK cells, and it was determined through the cytopathic effects, sequencing and electron microscopy. The informatics analysis of the *Fiber* was done using online bioinformatics servers. The CAdV-1-JL2021 strain was isolated successfully, and were most similar to the CAdV-1 strain circulating in Italy. The occurrence of negative selection and recombination were found in the CAdV-1-JL2021 and CAdV-2-AC_000020.1. Host cell membrane was its subcellular localization. The CAdV-1-JL2021 *Fiber* (ON164651) had 6 glycosylation sites and 107 phosphorylation sites, exerted adhesion receptor-mediated virion attachment to host cell, which was the same as CAdV-2-AC_000020.1 Fiber. The *Fiber* tertiary structure of the CAdV-1-JL2021 and CAdV-2-AC_000020.1 was different, but they had the same coxsackievirus and adenovirus receptor. “VATTSPTLTFAYPLIKNNNH” were predicted to be the potential CAdV-1 B cell linear epitope. The MHC-I binding peptide “KLGVKPTTY” were both presented in the CAdV-1-JL2021 and CAdV-2-AC_000020.1 Fiber and it is useful to design the canine adenovirus vaccine.

## Introduction

Canine adenovirus type 1 (CAdV-1) is a double-stranded DNA genome virus belonging to the mastadenovirus genus of the adenoviridae family. This virus is the etiologic agent of fox encephalitis (Balboni et al., 2019). CAdV-1 is transmitted by saliva, feces, urine (Yang et al., 2019). Foxes infected with CAdV-1 present with fever, cough, depression, vomiting, diarrhea, anorexia, convulsions, and the characteristic clinical sign of blue eye (corneal edema) (Sun et al., 2019). The large-scale use of the CAdV-2 live attenuated vaccine has reduced the prevalence of CAdV-1. However, CAdV-1 also circulates in wildlife with high pathogenicity and a broad host range (Verin et al., 2019; Zhu et al., 2020). Surveillance for new genetic variants is necessary to evaluate the potential impacts of CAdV-1 on wildlife species.

The CAdV-1 genome is 32 kb in length and contains 30 open reading frames (Pizzurro et al., 2017). The *Fiber* protein is the main capsid protein. The *Fiber* protein is composed of 3 domains, referred to as the tail, shaft, and knob (Schoehn et al., 2008). During CAdV-1 replication, the *Fiber* protein binds to the receptor, destroys the binding integrity of the receptor, allowing for viral entry into the host cell, and subsequent dissemination between cells. This cell to cell spread helps the virus minimize time in the extracellular environment, greatly limiting detection by the immune system (Walters et al., 2002). When CAdV-1 is detected and identified, *Fiber* nucleotide sequences are usually selected for homology and similarity analysis in CAdV-1 and CAdV-2 (Balboni et al., 2022).

An immunogenic protein, the *Fiber* protein is an attractive candidate for use in the development of subunit vaccines (De Luca et al., 2020). *Fiber* protein subunit vaccines have been demonstrated to protect fowl against fowl adenovirus serotype 4 (Ruan et al., 2018) and FAdV-8b (Gupta et al., 2017). Limited data have been reported demonstrating efficacy of *Fiber* protein vaccines against CAdV-1. The bioinformatics may have important applications in the newly discovered and emerging viruses prediction (Foroutan et al., 2018). Bioinformatics analysis of the ROP8 protein was conducted during the design process of a vaccine targeting *Toxoplasma gondii* (Foroutan et al., 2018). Bioinformatic analysis of the *Fiber* will be the first step to design the vaccine.

In the present study, a CAdV-1 isolated from a sick fox on a farm was characterized and the resulting *Fiber* sequence data were analyzed using bioinformatics. The resulting data provides a rational and theoretical starting point for the development of CAdV-1 control measures and vaccine development.

## Material and Methods

### Ethics Statement

This experiment was approved by the ethics committee at the Jilin Agriculture Science and Technology University, and the procedures complied with IACUC guidelines on the animals’ care and use for scientific purposes.

### Preparation of Liver Tissue

A fox presenting with clinical encephalitis was collected from a farm in Jilin. One part of the liver was stained by hematoxylin and eosin (H&E) (Lin et al., 2018). The other part of the liver was homogenized in 1 mL of sterile phosphate buffer (PBS), and was centrifuged for 10 min at 8000 g/min. The supernatants were collected and filtered through a filter with a 0.22 µ M pore size and stored at - 80°C.

### Cytopathic Effect and Virus Morphology

CAdV-1 infection was performed in Madin-Darby Canine Kidney (MDCK) cells (ATCC CCL-34, derived from normal kidney of Canis familiaris). MDCK cells were cultured at 37°C in Dulbecco’s modified Eagle’s medium (DMEM) containing 10% of fetal bovine solution (FBS) and 1% Penicillin-Streptomycin in a 5% CO_2_ incubator. Confluent MDCK cells (70-90%) were washed with PBS (pH 7.2), and inoculated with 100 µL of filtered liver homogenate supernatant at 37°C for 1 h. Nine milliliters of fresh DMEM containing 5% FBS was added, and the plate was incubated at 37°C in 5% CO_2_. The cytopathic effect (CPE) was observed and photographed daily. The MDCK infected cells with distinct CPE were further characterized by negative-stain transmission electron microscopy (TEM) to determine morphological characteristics of the infecting virus (Prasad et al., 2020).

### Polymerase Chain Reaction

Following infection, DNA was extracted from MDCK cells using the MagicPure^®^ Simple 32 Viral DNA/RNA Kit (EC311-32-11). Amplification of the 508bp CAdV-1 fragment was accomplished using previously published primers (Hu et al., 2001) (Forward primer-HA1:5’-CGCGCTGAACATTACTACCTTGTC-3’ and Reverse primer*-*HA2:5’-CCTAGAGCACTTCGTGTCCGCTT-3’). Reactions were run in a 50 µL sample volume consisting of 1 µL of DNA template, 25 μL of 2×EasyTaq^®^ PCR SuperMix (Transgen Biotech, AS111-01), 1 µL of forward and reverse primers each, and 22 µL of sterile deionized nuclease free water. The thermocycling profile for amplification was as follow: 5 min of denaturation at 95°C followed by 35 cycles of 1 min of denaturation at 95°C, 15 s of annealing at 58°C, and 30 s of extension at 72°C, with a final 5 min of extension at 72°C. Amplicons were resolved using1.0% agarose gel electrophoresis and stained with GelStain (Transgen Biotech, GS101-01) to visualize the product.

PCR amplification using the CAdV-1 *Fiber* primer pair (Fiber-F:5’-ATGAAGCGGACACGAAGTGCT-3’; Fiber-R:5’-TCATTGATTTTCCCCCACATAGGTGAAG-3’) yielding a 1063 bp fragment. The target region PCR reactions were performed six times in 50 µL reaction volumes containing 1 µL denatured DNA template, 5 μL of buffer, 2 μL of 25mM MgSO4, 1 µL of each primer (10 pmol/µL), 5 μL of 2 mM dNTPs, 1 μL of KOD plus (TOYOBO, KOD-201, 1.0 U/µL) and 34 µL of sterile deionized water. The thermocycling profile used was as follows: 5 min denaturation at 95°Cfollowed by 30 cycles of 30 s denaturation at 95°C, 30 s annealing at 60°C, and 1 min extension at 72°C, with a final 5 min extension at 72°C. The amplicons were resolved using 1.0% agarose gel electrophoresis and stained with GelStain (Transgen Biotech, GS101-01).

### Phylogenetic and Homology Analyses

Electrophoretically resolved amplicons from the PCR reactions were eluted from the gel matrix using the EasyPure^®^ Quick Gel Extraction Kit (TransGen, EG101-01), and cloned into an amplification plasmid using the pMD™18-T Vector Cloning Kit (TaKaRa, 6011). Sequencing of the purified plasmids was performed using Sanger sequencing by the Comate Bioscience Co., Ltd. The sequences were assembled and aligned according to the CAdV-1 reference sequences in GenBank, and were translated into amino acid sequences using BioEdit 7.2.5. The resulting *Fiber* nucleotide sequence (CAdV-1-JL2021 strain) was blasted in the NCBI database and compared to the 14 most similar *Fiber* nucleotide sequence of all CAdV strains. Based on the sequences, a phylogenetic tree was constructed using the MEGA 7.0.20 software with the neighbor-joining method. The reliability of the phylogenetic tree was verified through the bootstrap method with 1,000 replicates.

### Selective Pressure and Recombination Analysis

Based on the CAdV-1-JL2021 and CAdV-2-AC_000020.1 Fiber nucleotide sequence, the selective pressure and recombination analysis were conducted through SLAC (Kosakovsky Pond and Frost, 2005) and GARD (Kosakovsky Pond et al., 2006) method in Datamonkey.

### Protein Subcellular Localization and Function Prediction

The Protein subcellular localization of CAdV-1-JL2021 *Fiber* and CAdV-2-AC_000020.1 *Fiber* was predicted by Virus-mPLoc in Cell-PLoc 2.0 (Chou and Shen, 2008). The *Fiber* function of the CAdV-1-JL2021 and CAdV-2-AC_000020.1 were predicted by EMBL-EBI (Madeira et al., 2022).

### Bioinformatics Analysis of *Fiber* Protein

#### Physical and Chemical Properties, Phosphorylation, and Glycosylation Sites

Protparam (Walker, 2005) was used to predict the instability coefficient, average water absorption coefficient, relative molecular weight, amino acid composition and isoelectric point of charged amino acids in the *Fiber* protein. The Protscale with Hphob./Kyte & Doolittle (Walker, 2005) was used to predict the hydrophilicity and hydrophobicity of the *Fiber* protein.

The NetPhos 3.1 Server (Blom et al., 1999) (Residues to predict serine-all three, Output format-classical, Generate graphics) was used to predict the potential phosphorylation sites of the *Fiber* protein. The potential glycosylation sites of the *Fiber protein* were predicted using the NetNGlyc 1.0 Server (Gupta and Brunak, 2002) with generate graphics.

#### Transmembrane Region, Signal Peptide, Secondary and Tertiary Structure Prediction

In the CAdV-1-JL2021 Fiber, CAdV-2-AC_000020.1 *Fiber*, the transmembrane region was predicted using TMHMMServer 2.0 (Krogh et al., 2001) (Extensive, with graphics). SignalP 5.0 Server (Nielsen et al., 2019) (Eukarya, Long output) was used to predict the signal peptide. Potential secondary structure characteristics were predicted using SOPMA (Deléage ALIGNSEC, 2017) (Number of conformational states-4 (Helix, sheet, turn, coil), Similarity threshold-8, Window width-17). The tertiary structure of CAdV-1-JL2021 Fiber, CAdV-2-AC_000020.1 *Fiber* and the Coxsackievirus and adenovirus receptor _XP_038299179 were predicted using Robetta (Nerli and Sgourakis, 2019).

#### T and B Cell Epitope Prediction

The BCpred method (El-Manzalawy et al., 2008) (Methods: Fixed length epitope prediction- BCPred, Epitope length-20, Specificity-75%, report only non-overlapping epitopes) was used to predict potential B cell epitopes. MHC-I binding peptides were obtained using the IEDB Analysis Resource (Vita et al., 2019) (Sort peptides by-Predicted Score (descend), Output format-XHTML table). The MHC allele selected for the analysis was DLA-8803401, and the length was 14 aa, with the MHC source species being dog. Binding predictions of MHC-II performed using IEDB Analysis Resource as well. The prediction method used was IEDB recommended 2.22. The selected MHC allele was DRB1*01:01.

## Results

### Virus Isolation and Identification

**Figure 1:**
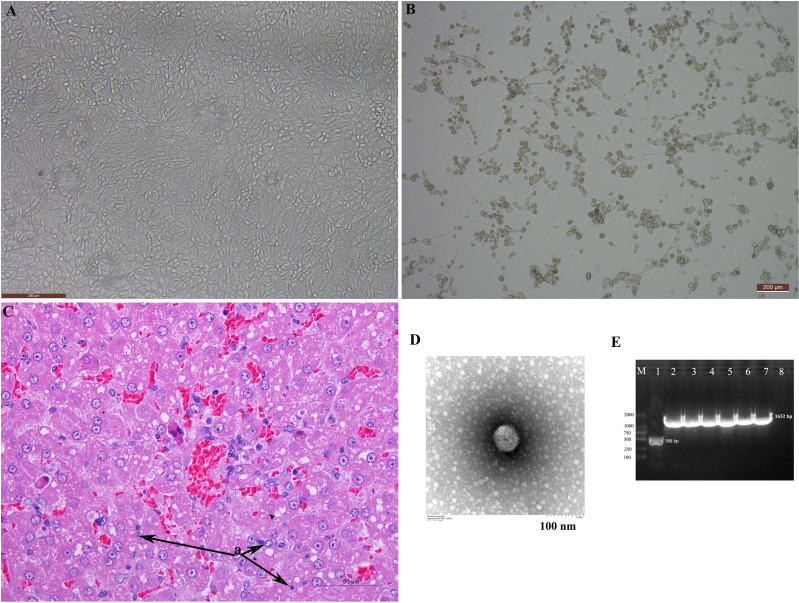
The isolation and identification of the canine adenovirus 1. **(A)** Normal MDCK; **(B)** MDCK with CPE; **(C)** HE staining of liver; **(D)** Electron microscopy observation of the adenovirus particles; **(E)** Amplification of the Fiber gene and identification of adenovirus.

The CPE was observed in MDCK cells. The typical “grape cluster morphology” of CPE was observed while control monolayers remained intact (**Figures 1A, B**). CAdV-JL2021 caused a series of morphological changes in fox liver, such as cell swelling and necrosis (**Figure 1C**). The cell with CPE were further examined by TEM, PCR, and sequencing. Characteristic adenoviral particles were observed by TEM (**Figure 1D**). A 508 bp fragment of the E3 gene was amplified for primary detection. It indicated that the CAdV-1 strain was first isolated. Next, a 1632 bp (**Figure 1E**) target band consistent with the *Fiber* gene was amplified and submitted to the GeneBank (ON164651).

### The Fiber Phylogenetic and Homology Analyses

**Figure 2:**
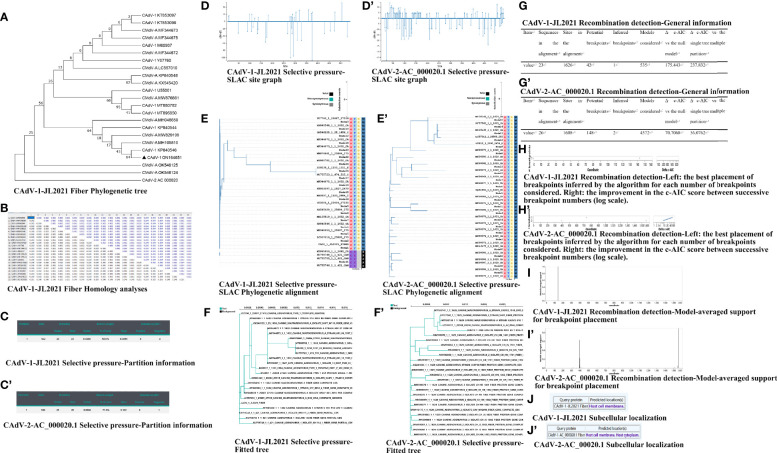
The nucleotide analysis of the Fiber. **(A)** CAdV-1-JL2021 Fiber Phylogenetic tree; **(B)** CAdV-1-JL2021 Fiber Homology analyses; **(C)** CAdV-1-JL2021 Selective pressure-Partition information; **(C’)** CAdV-2-AC_000020.1 Selective pressure-Partition information; **(D)** CAdV-1-JL2021 Selective pressure-SLAC site graph; **(D’)** CAdV-2-AC_000020.1 Selective pressure-SLAC site graph; **(E)** CAdV-1-JL2021 Selective pressure-SLAC Phylogenetic alignment; **(E’)** CAdV-2-AC_000020.1 Selective pressure-SLAC Phylogenetic alignment; **(F)** CAdV-1-JL2021 Selective pressure-Fitted tree; **(F’)** CAdV-2-AC_000020.1 Selective pressure-Fitted tree; **(G)** CAdV-1-JL2021 Recombination detection-General information; **(G’)** CAdV-2-AC_000020.1 Recombination detection-General information; **(H)** CAdV-1-JL2021 ecombination detection-Left: the best placement of breakpoints inferred by the algorithm for each number of breakpoints considered. Right: the improvement in the c-AIC score between successive breakpoint numbers (log scale). **(H’)** CAdV-2-AC_000020.1 Recombination detection-Left: the best placement of breakpoints inferred by the algorithm for each number of breakpoints considered. Right: the improvement in the c-AIC score between successive breakpoint numbers (log scale). **(I)** CAdV-1-JL2021 Recombination detection-Model-averaged support for breakpoint placement; **(I’)** CAdV-2-AC_000020.1 Recombination detection-Model-averaged support for breakpoint placement. **(J)** CAdV-1-JL2021 Subcellular localization; **(J’)** CAdV-2-AC_00020.1 Subcellular localization.

Three different clusters were identified through the phylogenetic analyses of the *Fiber* gene sequences accessioned in GenBank. The CAdV-1-JL2021 strain belongs to CAdV-1, and apparently has a close relationship with CAdV-1 strain (accession number, KP840546). This present study revealed the unique nature of the new strain (**Figure 2A**).

The protein sequences of the CAdV-1 *Fiber* gene (1632 bp in length, 543 amino acid residues) were compared with CAdV *Fiber* gene sequences available from NCBI (**Figure 2B**). The sequence of the *Fiber* gene was determined and was more similar with isolates from Italy (KP840546), suggesting that the isolated virus was CAdV-1. The virus identified in this study also shared 97.8 to 99.82% identity with other CAdV-1 strains.

### The Fiber Selective Pressure Analysis

Negative selective pressure was identified in *375, 281, 292, 170 sites of* CAdV-1-JL2021 Fiber (**Figures 2C–F**). Negative selective pressure was identified in *22 sites of* CAdV-2-AC_000020.1 Fiber (**Figures 2C’–F’**).

### The Fiber Recombination Analysis

The evidence of recombination breakpoint was found in the CAdV-1-JL2021 and CAdV-2-AC_000020.1Fiber GARD analysis. The CAdV-1-JL2021 alignment contained 42 potential breakpoints and 1 inferred breakpoint (**Figures 2G–I**). The CAdV-2-AC_000020.1 alignment contained 148 potential breakpoints and 2 inferred breakpoint (**Figures 2G’–I’**).

### *Fiber* Protein Subcellular Localization and Function Prediction

The predicted location of the CAdV-1-JL2021 *Fiber* was host cell membrane (**Figure 2J**). The predicted location of the CAdV-2-AC_000020.1 *Fiber* was host cell membrane and host cytoplasm (**Figure 2J’**). CAdV-1-JL2021 and CAdV-2-AC_000020.1*Fiber* exerted adhesion receptor-mediated virion attachment to host cell.

### *Fiber* Prediction of Physical and Chemical Properties

**Figure 3:**
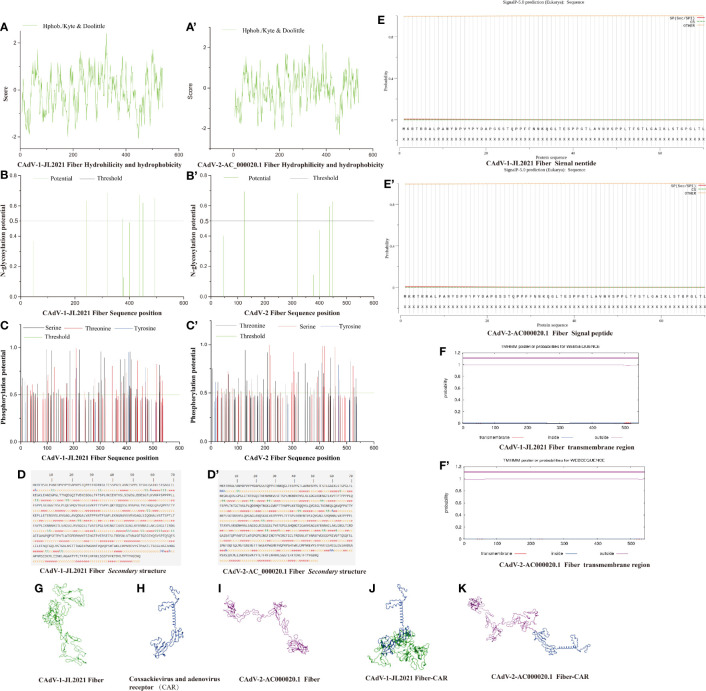
The structure prediction of the Fiber protein. **(A)** CAdV-1 JL2021 Fiber Hydrohilicity and hydeophobicity; **(A’)** CAdV-2-AC_000020.1 Fiber Hydrohilicity and hydeophobicity; **(B)** CAdV-1 JL2021 Fiber N-glycosylation potential position; **(B’)** CAdV-2-AC_000020.1 Fiber N-glycosylation potential position; **(C)** CAdV-1 JL2021 Fiber Phosphorylation potential position; **(C’)** CAdV-2-AC_000020.1 Fiber Phosphorylation potential position; **(D)** CAdV-1 JL2021 Fiber secondary structure; **(D’)** CAdV-2-AC_000020.1 Fiber secondary structure; **(E)** CAdV-1 JL2021 Fiber Signal peptide; **(E’)** CAdV-2-AC_000020.1 Fiber Signal peptide; **(F)** CAdV-1 JL2021 Fiber transmembrane region; **(F’)** CAdV-2-AC_000020.1 Fiber transmembrane region; **(G)** CAdV-1 JL2021 Fiber Three-dimensional structure; **(H)** Coxsackievirus and adenovirus receptor (CAR) Three-dimensional structure; **(I)** CAdV-2-AC_000020.1 Fiber Three-dimensional structure; **(J)** Interaction between CAdV-1 JL2021 Fiber and CAR; **(K)** Interaction between CAdV-2-AC_000020.1 Fiber and CAR.

The number of amino acids in the *Fiber* protein was 543, the molecular weight was 56974.49 g/mol, the isoelectric point was 6.26, and the instability coefficient was 39.33. There were 143 amino acids in the hydrophobic region (> 0.5), 168 amino acids in the hydrophilic region (< - 0.5), suggesting that CAdV-1-JL2021 *Fiber* was a hydrophilic protein. (**Figure 3A**). According to the CAdV-2-AC_000020.1 *Fiber*, the average hydrophobicity of the *Fiber* protein is -0.092. There were 129 amino acids in the hydrophobic region (> 0.5), 161 amino acids in the hydrophilic region (< - 0.5), suggesting that CAdV-2-AC_000020.1 *Fiber* was a hydrophilic protein (**Figure 3A’**).

### *Fiber* Prediction of Phosphorylation and Glycosylation Sites

In **Figure 3B**, 6 glycosylation sites (242, 319, 375, 438, 450 and 493) were identified in the *Fiber* protein. It can be seen in **Figure 3C**. CAdV-1-JL2021 *Fiber* had 59 serine phosphorylation sites, 42 potential threonine phosphorylation sites and 6 tyrosine phosphorylation sites. In **Figure 3B’**, 4 glycosylation sites (125, 320, 437, 449) were identified in the *Fiber* protein. It can be seen in **Figure 3C’**, CAdV-2-AC_000020.1 *Fiber* had 42 serine phosphorylation sites, 47 potential threonine phosphorylation sites and 8 tyrosine phosphorylation sites. However, the *Fiber* protein in CAdV-1-JL2021 and CAdV-2-AC_000020.1 were not expected to have either a transmembrane region or signal peptide, as were shown in **Figures 3E, E’, F, F’**, respectively.

### Prediction on *Fiber* Secondary and Tertiary Structure

The CAdV-1-JL2021 *Fiber* protein α- Helix (Hh), extended chain (EE), β- proportions of angle (TT) and irregular curl (CC) were 0.92%, 34.81%, 6.45% and 57.83% respectively (**Figure 3D**). The CAdV-2-AC_000020.1 *Fiber* protein α- Helix (Hh), extended chain (EE), β- proportions of angle (TT) and irregular curl (CC) were 1.29%, 36.53%, 4.24% and 57.93% respectively (**Figure 3D’**). The *Fiber* tertiary structure of the CAdV-1-JL2021 and CAdV-2-AC_000020.1 was different, but they had the same coxsackievirus and adenovirus receptor (**Figures 3G–I**). Their binding models were also predicted (**Figures 3J, K**).

### T and B Cell Epitope Prediction in *Fiber*

**Figure 4:**
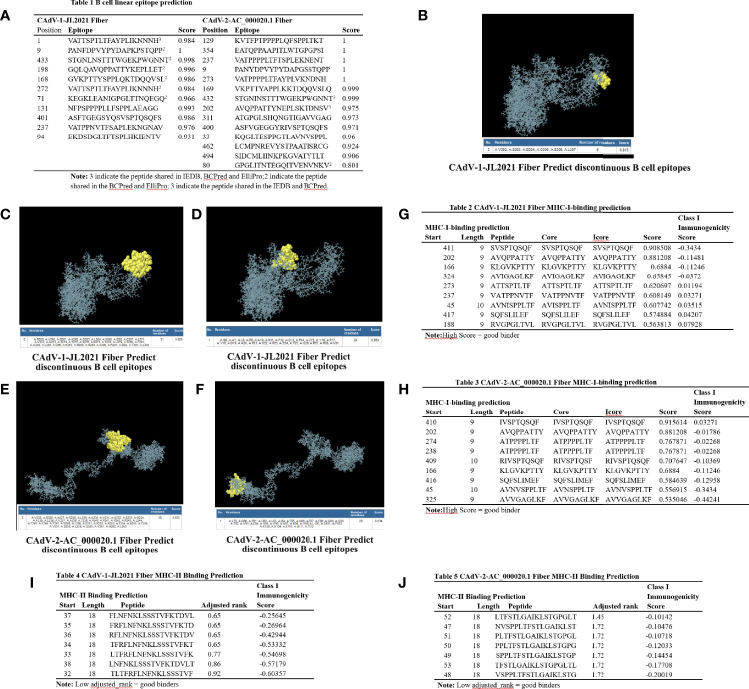
The epitope analysis of the Fiber protein. **(A)** Table 1 B cell linear epitope prediction; **(B–D)** CAdV-1 JL2021 Fiber discontinuous epitopes; **(E, F)** CAdV-2-AC_000020.1 Fiber discontinuous epitopes; **(G)** Table 2 CAdV-1-JL2021 Fiber MHC-I-binding prediction; **(H)** Table 3 CAdV-2-AC_000020.1 Fiber MHC-I-binding prediction. **(I)** Table 4 CAdV-1-JL2021 Fiber MHC-II Binding Prediction; **(J)** CAdV-2-AC_000020.1 Fiber MHC-II Binding Prediction.

11 and 14 of B cell linear epitopes were predicted in the *Fiber* of CAdV-1-JL2021 and CAdV-2-AC_000020.1, respectively (**Figure 4A**). The peptides in the 1, 9, 433, 198, 168, 272, 71 sites of CAdV-1-JL2021 were simultaneously predicted. Importantly, the peptide “VATTSPTLTFAYPLIKNNNH” was both predicted by the IEDB, BCPred and ElliPro. The peptides in the 432, 202, 80 sites of CAdV-2-AC_000020.1 were simultaneously predicted. CAdV-1-JL2021 and CAdV-2-AC_000020.1 Fiber did not have the same B cell linear epitopes. CAdV-1-JL2021 and CAdV-2-AC_000020.1 *Fiber* had 3 and 2 discontinuous epitopes, respectively (**Figures 4B–F**).

CAdV-1-JL2021 and CAdV-2-AC_000020.1 *Fiber* had 9 MHC-I binding peptides, respectively. Importantly, the peptide “KLGVKPTTY” were both presented in the CAdV-1-JL2021 and CAdV-2-AC_000020.1 *Fiber* (**Figures 4G, H**). CAdV-1-JL2021 and CAdV-2-AC_000020.1 *Fiber* had 7 MHC-II binding peptides, respectively (**Figures 4I, J**).

## Discussion

The size and shape of CAdV particles were similar to those of CAdV-1 and CAdV-2 strains isolated from fox (Choi et al., 2014; Tamukai et al., 2020). However, virus isolation is a very time-consuming diagnostic test, which further necessitates additional molecular tests to classify the etiologic agent as either CAdV-1 or CAdV-2. The haemagglutination and neutralization tests do not provide definitive differentiation between CAdV-1 and CAdV-2 isolated from the digestive tract (Timurkan et al., 2018). However, PCR is a powerful tool for the differentiation of CAdV-1 and CAdV-2 (Balboni et al., 2019; Oleaga et al., 2021). The genomic region encoding the E3 gene and flanking sequences were selected as the target for a pair of primers to diagnose and differentiate the two serotypes of CAdV (Hu et al., 2001). The resulting PCR product produced bands 508 bp of CAdV-1 E3 gene, and 1632 bp of CAdV-1 *Fiber* gene, which will identify the occurrence of CAdV-1 in fox using sequencing and phylogenetic analysis.

Double-stranded DNA viruses tend to have lower mutation rates than RNA genome viruses (Sanjuán and Domingo-Calap, 2016). Nucleotide sequence alignment between the CAdV-1-JL2021 strain and 14 reference strains showed high identities ranging from 97.8 to 99.82%. A phylogenetic tree of 23 *Fiber* nucleatide acid sequences found that the CAdV-1-JL2021 strain was included in the KP840546 strain containing subgroup. The study of this virus strain can provide an alternative strain for the diagnosis and prevention of fox encephalitis.

Selection is an essential component of any evolutionary system and analyzing this fundamental force in evolution can provide relevant insights into the evolutionary development of a population (Haasdijk and Heinerman, 2018). negative selection removed amino-acid changes that reduced fitness, positive selection maintained amino-acid changes that increase virus fitness. Neutral selection was that the fitness was not affected by the amino-acid changes. In this experiment, negative selective pressure was identified in *375, 281, 292, 170 sites of* CAdV-1-JL2021 Fiber and in the 22 sites of CAdV-2-AC_000020.1. The presence of negative selection implies that the sites were functionally important in the virus evolution. *The sites in the* CAdV-1-JL2021 *Fiber* were less than that of CAdV-2-AC_000020.1. *The less negative selection site maybe that* the numbers of nucleotide substitutions (cS 1 cN) observed were insufficient for detecting statistically significant differences between cS and cN. More sequence data should be collected in the future. The negative selection identified in CAdV-1-JL2021 and CAdV-2-AC_000020.1 protein may help the identification of highly conserved regions useful to implement new future diagnostic protocols.

Natural recombination is an important strategy for viruses to adapt to new environmental conditions and hosts. Besides evolving through nucleotide substitution, viruses frequently also evolve by genetic recombination which can occur when related viral variants co-infect the same cells (Varsani et al., 2018). More CAdV genome sequences were added to GenBank, CAdV-1 and CAdV-2 sequences arising from different parts of world. Therefore, it is necessary to find the recombination for genetic variability, and it will be helpful to understand the evolutionary process of the CAdV genome (Eltahir et al., 2011). In our experiment, recombination break-point were observed in the 143 site of CAdV-1-JL2021 *Fiber, and 397, 1558 site of* CAdV-2-AC_000020.1. In the evolution of the CAdV-1 populations, the recombination was not a common event. The genetic diversity of CAdV-1 evolutionary maybe attribute to the recombination. During the infection, the same animal maybe infected by different CAdV genotypes, and this condition will contribute to the CAdV recombination.

The subcellular location of a protein is highly related to its function (Pan et al., 2020). Identifying the location of a given protein is an essential step for investigating its related function. The host cell membrane was the subcellular location of the CAdV-1-JL2021 and CAdV-2-AC_000020.1. But host cytoplasm was also the subcellular location of the CAdV-2-AC_000020.1. It indicates that they may have different function during the virus infection. It predicted that CAdV-1-JL2021 and CAdV-2-AC_000020.1*Fiber* exerted adhesion receptor-mediated virion attachment to host cell. More evidences need to further find the function difference between the CAdV-1 and CAdV-2.

In the process of CAdV infection, the *Fiber* protein interacts with the host cell receptor to adsorb on the host cell. While much of the current literature focuses on analysis of the *Fiber* gene nucleic acid sequence of virus isolates (Schoehn et al., 2008), few have gone so far as to predict the molecular characteristics of the *Fiber* protein. The isoelectric point was predicted to be 6.26. When pH of the environment was 6.26, the net surface charge of CAdV-1-JL2021 *Fiber* protein was 0. At this time, the *Fiber* proteins repeled each other in solution, the force between molecules was weakened, and *Fiber* precipitation occured readily. Thus, at the isoelectric point, *Fiber* protein was easy to obtain because of its low solubility. When the instability coefficient of the protein was greater than 40, it was considered to be an unstable protein (Dong et al., 2021). The total average hydrophobicity of the hydrophilic protein was less than 0 (Zhou, 2016). The average hydrophobicity of the CAdV-1-JL2021 *Fiber* protein was -0.092, suggesting that it was a hydrophilic protein. Protein dissolution in supernatant was an ideal result of prokaryotic expression, but the specific distribution of protein in supernatant and precipitation still need to be analyzed by SDS-PAGE. In the prokaryotic expression and purification of *Fiber* protein, the corresponding test scheme can be formulated according to its hydrophilic characteristics.

Protein phosphorylation is crucial for multiple biological processes including signal transduction, regulation of cell cycle and gene expression through post-translational modifications (Luo et al., 2019; Mehrpouyan et al., 2021). For example, the N protein phosphorylation impairs porcine reproductive and respiratory syndrome virus growth efficiency in porcine alveolar macrophages (Chen et al., 2019). Thus, it is important to predict protein phosphorylation sites in the *Fiber* protein. It was predicted that CAdV-1-JL2021 *Fiber* protein had 107 phosphorylation sites. The phosphorylation of *Fiber* protein is related to its antigenicity and virulence. Like phosphorylation, glycosylation is also an important post-translational modification, which affects the structure and function of proteins (Macedo-da-Silva et al., 2021). The glycosylation of the Newcastle disease virus (Kosakovsky Pond et al., 2006) resulted in a virus that was able to proliferate faster than that of the vaccine (Schön et al., 2021). The CAdV-1-JL2021 *Fiber* protein has six glycosylation sites, suggesting that it is related to the virus titer and proliferation rate of CAdV-1. The phosphorylation sites and glycosylation sites of CAdV-1-JL2021 *Fiber* protein is the same as that of CAdV-2-AC_000020.1 *Fiber* protein. It indicates that they have the same function.

The secondary structure prediction results showed that the extended chain and irregular curl accounted for the majority of the *Fiber* protein secondary structure. The irregular curl is responsible for a protein’s enzymatic activity and protein specific functions (Simm et al., 2021). It was also observed that the CAdV-1-JL2021 and CAdV-2-AC_000020.1 *Fiber* protein contains a large number of antigenic determinants, which is consistent with the prediction results of antigenic determinants. Antigenic determinants can specifically bind to corresponding antibodies or B cells. Since the *Fiber* protein is the main capsid protein on the adenovirus surface, its antigenic determinants can cause strong neutralization reactions. Therefore, predicting the antigenic determinants of *Fiber* protein and the dominant B cell epitopes is helpful to understand the mechanisms of viral neutralization *via* responses to the *Fiber* protein.

The Knob of the adenovirus *Fiber* protein is used for attachment of the virus to a specific receptor on the cell surface. The coxsackievirus and adenovirus receptor (CAR) was both a viral receptor and cell adhesion protein (Readler et al., 2019). CAV-2 had been shown to use CAR as a primary receptor (Soudais et al., 2000). But limited data showed the interaction between Knob of the *Fiber* and the CAR. The CAdV-1 and CAdV-2 fiber–knobs may share a common receptor as evidenced by the ability of the CAdV- 1 knob to inhibit Ad5Luc1-CK2 function (Stoff-Khalili et al., 2005). In our experiment, The *Fiber* in CAdV-1-JL2021 and CAdV-2-AC_000020.1, CAR were predicted. The binding model between *Fiber* and CAR was also predicted. It will be helpful to clarify the interaction between *Fiber* and CAR.

The B cell linear peptide “VATTSPTLTFAYPLIKNNNH” in CAdV-1-JL2021 was predicted by the IEDB, BCPred and ElliPro. It indicates that this peptide had high potential in the epitope vaccine design. The B cell linear peptides “STGNINSTTTWGEKPWGNNT” of CAdV-2-AC_000020.1 were both predicted by the IEDB and BCPred. CAdV-1-JL2021 and CAdV-2-AC_000020.1 *Fiber* did not have the same peptide. It indicates that B cell linear epitope vaccine should be developed in the CAdV-1-JL2021 and CAdV-2-AC_000020.1, respectively. Importantly, the T cell peptide “KLGVKPTTY” were both predicted in the CAdV-1-JL2021 and CAdV-2-AC_000020.1 Fiber. It indicates that T cell epitope vaccine maybe both efficient for the CAdV-1-JL2021 and CAdV-2-AC_000020.1, respectively.

## Conclusion

In this experiment, the CAdV-1-JL2021 strain was isolated successfully, and were most similar to the CAdV-1 strain circulating in Italy. The occurrence of negative selection and recombination were found in the CAdV-1-JL2021. Host cell membrane was its subcellular localization. The CAdV-1-JL2021 *Fiber* (ON164651) exerted adhesion receptor-mediated virion attachment to host cell, which was the same as CAdV-2-AC_000020.1 Fiber. “VATTSPTLTFAYPLIKNNNH” were predicted to be the potential CAdV-1 B cell linear epitope. The MHC-I binding peptide “KLGVKPTTY” were both predicted in the CAdV-1-JL2021 and CAdV-2-AC_000020.1 *Fiber*, and it is useful to design the canine adenovirus epitope vaccine.

## Data Availability Statement

The original contributions presented in the study are included in the article/supplementary files. Further inquiries can be directed to the corresponding author.

## Ethics Statement

This experiment was approved on April 2 in 2021 by Jilin Agriculture Science and Technology College and the procedures complied with IACUCS guidelines on the animals’ care and use for scientific purposes.

## Author Contributions

BW and MW conduct the experiment. HZ, JX, and JH analyze the data and draw the picture. YZ designed and provide the grant for this experiment. YZ also reviews the manuscript. All authors contributed to the article and approved the submitted version.

## Funding

This work was supported by grants from the Science and Technology department of Jilin Province (20200402045NC) and the Doctoral Start-up Fund Project of the Jilin Agriculture Science and Technology College (20200002).

## Conflict of Interest

The authors declare that the research was conducted in the absence of any commercial or financial relationships that could be construed as a potential conflict of interest.

## Publisher’s Note

All claims expressed in this article are solely those of the authors and do not necessarily represent those of their affiliated organizations, or those of the publisher, the editors and the reviewers. Any product that may be evaluated in this article, or claim that may be made by its manufacturer, is not guaranteed or endorsed by the publisher.
